# Intrathecal Catheterization by Epidural Catheter: Management of Accidental Dural Puncture and Prophylaxis of PDPH

**Published:** 2009-02

**Authors:** Ashok Jadon, Swastika Chakraborty, Neelam Sinha, Rajiv Agrawal

**Affiliations:** 1 Senior Consultant and Head of the Department, Department of Anaesthesia, Tata Motors Hospital, Jamshedpur-831001, Jharkhand, (India); 2 Consultant, Department of Anaesthesia, Tata Motors Hospital, Jamshedpur-831001, Jharkhand, (India); 3 Senior Registrar, Department of Anaesthesia, Tata Motors Hospital, Jamshedpur-831001, Jharkhand, (India); 4 Senior Medical Officer, Department of Anaesthesia, Tata Motors Hospital, Jamshedpur-831001, Jharkhand, (India)

**Keywords:** Epidural anaesthesia, Accidental dural puncture, Post dural puncture headache (PDPH), Intrathecal catheter

## Abstract

**Summary:**

Accidental or inadvertent dural puncture during epidural anaesthesia results in high incidence of post dural puncture headache (PDPH). Spinal or intrathecal catheter in such a situation, provides a conduit for administration of appropriate local anaesthetic for rapid onset of intraoperative surgical anaesthesia and postoperative pain relief. This procedure prevents PDPH if catheter left in situ for > 24 hrs and also avoids the associated risks with a repeat attempts at epidural analgesia.

Primary aim of this study was to observe the effect of spinal catheter on incidence of PDPH, and to assess early and delayed complications of spinal catheterization by epidural catheter.

In prospective clinical study 34 patients who had accidental dural puncture during epidural anaesthesia were included. The catheter meant for epidural use was inserted in spinal space and used for spinal anaesthesia and postoperative analgesia. Catheter was removed between 24-36hrs after surgery.

The incidence of accidental dural puncture was 4%(34/846). Two patients 5.88% (2/34) had transient paresthesia during spinal catheter insertion. Post dural puncture headache occurred in 11.76% (4/34) patients. Two patients required epidural blood patch and two patients were managed with conservative treatment. No patient had any serious intraoperative or postoperative side effects.

Epidural catheter can be used as spinal catheter to manage accidental dural puncture without serious complications, and it also prevents PDPH.

## Introduction

Accidental dural puncture during epidural occurs in 0.4 to 6.0% patients.^1^ Due to large opening and associated CSF leak, large number of patients (75-86%) develop post-dural puncture headache (PDPH), which is mostly severe in nature.^2^ Epidural blood patch is an effective treatment of severe post-dural puncture headache however, its effectiveness decreases if dura mater puncture is caused by a large bore needle.^3^ Epidural catheters have been used to prevent severe PDPH and manage accidental dural puncture by insertion of epidural catheter in to the subarachnoid space and using them as spinal catheter for continuous spinal anaesthesia.^4^,^5^ Intrathecal catheter insertion following unintentional dural puncture reduces the requirement for epidural blood patch.^6^

Thirty-four cases intended for epidural anaesthesia were managed successfully with epidural catheter inserted in subarachnoid space after accidental dural puncture.

This is a prospective clinical study of thirty-four patients who had inadvertent dural puncture during epidural or combined spinal epidural anaesthesia between 1.1.2004 to 15.5.08. Hospital Ethical committee approval and informed (written) consent was taken from all the patients before procedure.

Technique: Epidural space was identified by loss of resistance to air in all cases with due asepsis. If inadvertent dural puncture occurred, epidural catheter (18G or 20G) was inserted 2-3 cm in subarachnoid space by one of the senior anaesthetist (one of the authors), who had previous experience of spinal catheterization and more than 15 years experience in anaesthesia. Catheter position was confirmed by easy aspiration of clear CSF and initial dose of 0.5% heavy bupivacaine (0.5-1.0ml) was given. Catheter was fixed and level of anaesthesia was assessed. Increments of 0.5% heavy bupivacaine was given as and when required.

Spinal catheters were removed between 24-36 hrs postoperatively. Patients were observed for 7 days in the hospital and later on follow-up was done either through concerned surgeon during patient's visit to the hospital or by personal communication. Intraoperative data was collected regarding patients profile, probable cause of dural puncture, type of surgery and immediate complication during or after catheter insertion. Post-operatively data was collected regarding total doses of intrathecal drugs, post-spinal headache and any other complications. Patients who developed PDPH were managed with parenteral fluids, analgesic drugs and epidural blood patch.

## Results

During 1.1.2004 to 15.5.08 accidental dural puncture was noticed in 34 (3.8%) patients out of 885 patients scheduled for various surgeries under epiduralor combined spinal epidural anaesthesia (Table-1 & Table-2). The possible causes of accidental dural puncture are shown in Table-3; anatomical difficulty and repeated attempts were responsible for dural puncture in 17 (50%) of the patients. Unable to identify loss of resistance at epidural space in 6 (17.6%), turning the bevel after localization of epidural space to facilitate catheter insertion in 6 (17.6%), and Sudden movement of patient in 5 (14.7%) patients were the causes of accidental dural puncture in remaining half of the patients. Outcome and complications are shown in Table-4; insertion of epidural catheter in subarachnoid space was easily done in 32(94.11%) patients, and redirection during insertion was required in 2 (5.88%) patients due to paresthesia on initial insertion. All the catheters were left in subarachnoid space for more than 24 hrs (mean 28.85 ± 3.92 hrs), and 4(11.76%) patients developed post dural puncture headache (PDPH) after catheter removal. Epidural blood patch was offered to four patients who developed PDPH after catheter removal. Two patients refused and were managed conservatively by Tab paracetamol, diclofenac injections, intravenous fluids and bed rest. PDPH was cured after 4^th^ day and 7^th^ day consecutively in both patients. Epidural blood patch was given by 15 ml in one patient and 18ml in second patient on the same day after catheter removal, relieved the PDPH instantaneously. Patients' who received epidural blood patch complained of moderate backache which was treated with tab. paracetamol and was relieved next day. No patient showed any sign of CSF leak, neurological injury or recurrence of PDPH. No complication of catheter knotting or infection either at the entry (exit) site or in deeper tissues was seen.

**Table 1:** Incidence of dural puncture, sex ratio and mean age of the patients

Total number of epidurals	885
Inadvertent dural puncture	34
Incidence	3.8%
Male Female ratio	20 female, 14 male
Mean age (SD)	47 (±17.67) years

**Table 2:** Types of surgical procedures and incidence of accidental dural puncture(n)

Types of surgical procedures	Accidental dural puncture	Number of cases
Abdominal hysterectomy	06	179
Hip # for decompression hip screw	04	134
Hip arthroplasty	05	128
Knee arthroplasty	04	100
Incisional hernia repair	04	111
Inguinal hernia repair	05	98
Laminectomy	01	08
Labour analgesia	02	18
L SC S	02	60
Cholecystectomy	01	49
Total	34	885

**Table 3:** Possible cause of dural puncture during epidural precedure(n)

Difficult anatomy requiredmore than one attempt	17
Unable to identify loss of resistance at epidural space	06
Sudden movement of patient	05
Turning the bevel after localization of epidural space	06
Total	34

**Table 4:** Out come and complications of subarachnoid catheter insertion and catheter removal

Easy insertion	32(94.11%)
Paresthesia (required redirection)	02(5.88%)
Mean duration of spinal catheter in situ (Mean hrs. ±SD)	28.85(±3.92)
Number of patients with PDPH (%)	04/34(11.76)
Number of patients managed conservatively	02*
Number of patients required epidural blood patch	02**
Infection at entry (exit) site	Nil
Deep tissue infection	Nil
Catheter tip infection	Nil
CSF leak	Nil
Meningitis	Nil
Other neurological complication (Paresthesia, Bladder or Bowel dysfunction)	Nil
Knotting of catheter either during insertion or removal	Nil

* Managed with oral fluids, paracetamol and inj. diclofenac.

** Both the patient experienced backache for 24 hrs.

## Discussion

Incidence of accidental dural puncture during epidural varies 0.4% to 6.0%^1^, we had incidence of 3.84% (Table-1) which is well with in range. However, it was on the higher side of mean. It may be because heterogeneous group of patients (Table-2), difference in experience of authors and method used for epidural localization. Accidental dural perforation is inversely proportional to anaesthesiologists' experience^7^ and saline is better than air for epidural localization.^8^ Repeated attempts have been associated with increased accidental dural puncture.^9^ In present study the highest incidence of accidental dural puncture occurred during repeated attempts for epidural (Table-3), either due to difficult anatomy or anxious, uncooperative patients. In our study four patients [11.76%(4/34)] developed PDPH after removal of epidural catheter. Two patients were having mild symptoms and were managed with increased oral fluid intake and analgesics (Tablet paracetamol and diclofenac).

An epidural blood patch (EBP) remains the standard against which all other treatments for a PDPH are compared however it is not without its complications. Back pain, neck pain, leg pain and paresthesias have been reported following the administration of an EBP.^10^ Two obstetric patients who did not respond to analgesics were given epidural blood patch (15ml and 18 ml) and PDPH was cured. Both the patients had moderate backache which resolved over next 24 hrs.

Paresthesia during catheter insertion is not uncommon but transient paresthesias are mostly benign.^11^ Two patients of our study had paresthesia during catheter insertion, which resolved with redirection of catheter. No postoperative side effect was noticed. In our study all the thirty-four patients who had accidental dural puncture were managed by spinal catheter and no intraoperative or serious postoperative complications occurred. The continuous spinal anaesthesia is a standard anaesthesia technique, although the use of epidural catheter as spinal catheter was debatable.^12^,^13^ The use of epidural catheter as spinal catheter got acceptance slowly as it was noted that thin catheter which were meant for continuous spinal anaesthesia were actually responsible for neural complications and their insertion is also difficult.^14^,^15^ More and more data is now becoming supportive of spinal catheterization.^16^,^17^ Deliberate intrathecal insertion of an epidural catheter after accidental dural puncture has been reported in obstetric patients and found to be effective in prophylaxis of PDPH.^18^ The incidence of PDPH in our study was 11.76% which is similar to other catheter related studies,^16^–^18^ and this incidence is far less than the reported PDPH incidence of 46%-86% in cases of accidental dural puncture without prophylaxis.^2^

Etiology by which spinal catheter prevents PDPH is not known however it is postulated that it stimulates inflammatory cells to accumulate near the entry of catheter and closing of dural tear. Formation of fibrin around the intrathecal catheter at the dural tear has also been described in an experimental study using cats.^19^ This also explains that, why two of our patients responded to conservative treatment.

Insertion of epidural catheter in spinal space after accidental dural puncture is now becoming common. A survey in the United Kingdom aimed to explore the current management of accidental dural puncture compared the findings to a similar survey undertaken in 1993. In 47 units (28%), the epidural catheter is now routinely placed in trathecally following accidental dural puncture. This is in contrast to the previous survey, which found that catheters were re-sited in 99% of units.^20^ Our study included cases from all specialties and therefore there is large variation in nature of surgery. Subarachnoid catheter placement after wet tap in obstetric patients reduces the PDPH rate and does so to a greater extent if left in place for 24hours.^21^ That's why our target to keep the spinal catheter was > 24 hrs however, if time of removal was commencing at late evening; the catheters were removed in the next morning. Therefore duration of catheter in situ is also quite variable because practically it was very difficult to remove catheter exactly after 24 hrs.

Epidural catheter can be used as spinal catheter to manage accidental dural puncture during epidural anaesthesia. If catheter left in situ for 24-36 hrs provides prophylaxis against PDPH. In our experience of 34 cases of accidental dural puncture, we found it is safe to practice. However, a large double blind randomized trial is necessary to prove its ultimate safety.
